# Gender difference in advanced HIV disease and late presentation according to European consensus definitions

**DOI:** 10.1038/srep14543

**Published:** 2015-09-28

**Authors:** Hongbo Jiang, Jieyun Yin, Yunzhou Fan, Jianhua Liu, Zhixia Zhang, Li Liu, Shaofa Nie

**Affiliations:** 1 Department of Epidemiology and Biostatistics, School of Public Health, Tongji Medical College, Huazhong University of Science and Technology, 430030 Wuhan, Hubei, P.R. China

## Abstract

Effectiveness of highly active antiretroviral therapy is limited for a large proportion of individuals living with HIV presenting for medical care at an advanced stage. Controversial results of gender differences in risk of late HIV diagnosis were reported among existing literatures. Therefore, we conducted this meta-analysis to synthesize a summary of gender differences in risk of advanced HIV disease (AHD) and late presentation (LP) according to European consensus definitions. Totally, 32 studies were included based on predetermined selection criteria. The pooled adjusted *odds ratios* of males presenting with AHD and LP compared with females were 1.73 (*95*% *confidence interval [CI]*, 1.59–1.89) and 1.38 (*95*% *CI*, 1.18–1.62) with significant heterogeneity observed (*I*^*2*^ = 78.50%, and *I*^*2*^ = 85.60%, respectively). Subgroup analysis revealed that time lag, study location, number of patients, proportion of females, study design, number of adjusted variables might be potential source of heterogeneity. Sensitivity analysis showed robustness of the results. No publication bias was observed in studies on AHD or LP. The current meta-analysis indicated that males are at higher risk of AHD or LP compared with females. More attention should be paid to males to make sure early testing, diagnosis, and treatment, and ultimately improve individual and population health.

Highly active antiretroviral therapy (HAART) has dramatically reduced HIV-related morbidity and mortality since it has been available in the mid 1990s. Many HIV infected individuals are living with HIV/AIDS as a chronic manageable disease rather than an inevitably progressive and fatal illness^1^,^2^. However, the effectiveness of HAART at the individual and population level is limited for a large proportion of individuals living with HIV presenting for medical care at an advanced stage, thereby missing the opportunity for the timely beginning of HAART^3^,^4^.

Late presenters are associated with higher rates of opportunistic infections^5^,^6^, lower virologic and immunologic efficacy of HAART^7^,^8^,^9^, shorter survival^10^, and increased HIV-related morbidity and mortality^6^,^10^,^11^,^12^ from an individual perspective. Late presentation also contributes to more complex treatment^13^ and higher health care costs^14^,^15^,^16^, and increased risk of HIV transmission due to unawareness of serostatus^17^,^18^,^19^ from a public health perspective. On the contrary, early diagnosis and subsequent timely treatment in the course of infection before severe impairment of the immune system increase life expectancy which may approach that of the general population and improve quality of life^20^,^21^.

However, different criteria have been used to define late presentation among HIV infected individuals, which generally include CD4 cell count and/or AIDS-defining diseases^22^. Then the European Presenter Consensus working group proposed common definitions of late HIV diagnosis that “late presentation” be defined as the presence of an AIDS condition or CD4 cell count <350 cells/μL at presentation for care, leaving the term “advanced HIV disease” to describe the presence of either an AIDS condition or a CD4 cell count <200 cells/μL at presentation^12^,^23^. Yet, an inconsistent short time period between initial diagnosis of HIV infection and AIDS diagnosis ranging from one month to twelve months, and even five years was used to define late HIV diagnosis in most previous studies, which makes examination of factors associated with late diagnosis difficult^24^.

Previous studies showed that males were more likely to present late in HIV diagnosis^25^,^26^, while some other studies presented no significant difference between males and females in late HIV diagnosis^27^,^28^. These conflicting reports are reflective of the difference in health-seeking behaviors and HIV testing policies, as well as the heterogeneity of the parameters used among the different studies. Thus, we performed a meta-analysis of all relevant published literature in order to provide a summary risk estimate of gender difference in advanced HIV disease (AHD) and late presentation (LP) according to European consensus definitions.

## Results

### Literature selection

**Figure 1:**
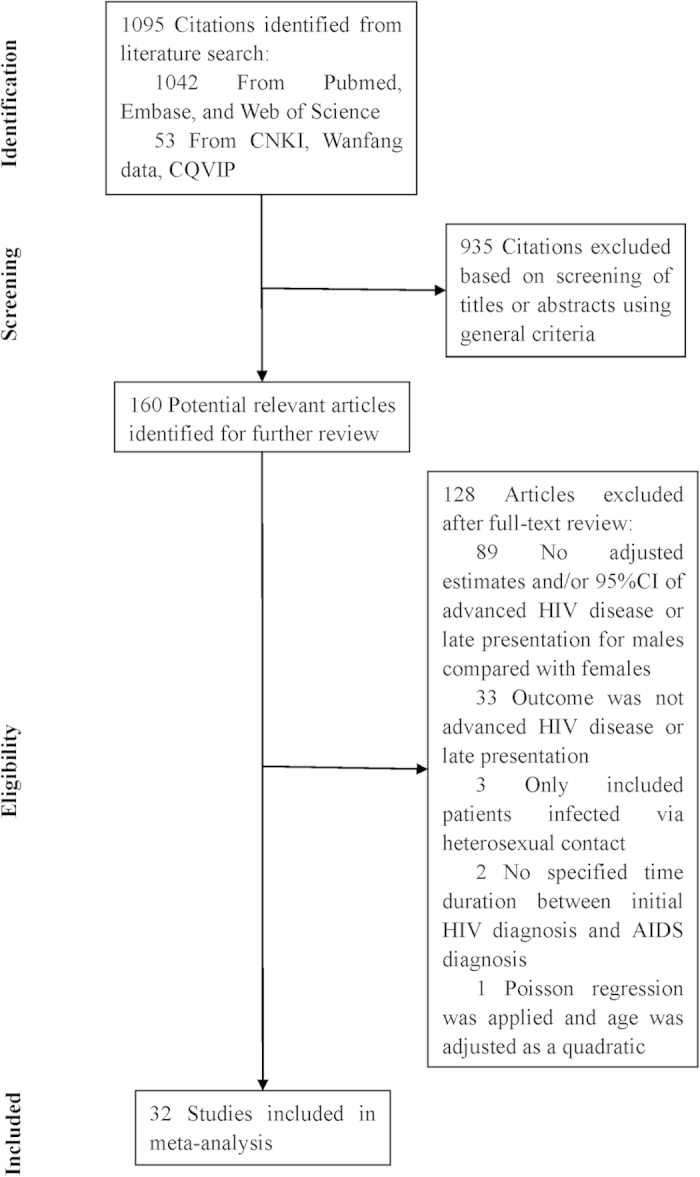
Flow chart of study selection.

The search strategy retrieved 1095 unique citations. Of these, 935 citations were excluded after the first screening based on abstracts or titles, leaving 160 articles for full-text review (Fig. 1). On this review, 89 articles without adjusted estimates or *95*% *confidence interval (CI)*, 33 articles with inconsistent definition of outcomes, 3 articles only recruited patients infected via heterosexual contact, 2 articles contained unspecified the exact duration between initial HIV diagnosis and AIDS diagnosis, were excluded. After this selection, only one study used Poisson regression adjusted by age as a quadratic to calculate adjusted *relative risk (aRR)*, while the others used logistic regression to calculate adjusted *odds ratios* (*aOR*). This study was finally excluded owing to small number and inconsistency in method, leaving 32 studies for final inclusion in the meta-analysis.

### Study characteristics

Characteristics of the 43 arms in 32 selected studies are shown in Supplementary Table S1. Of all, one study was prospective study, six studies were cross-sectional studies, and the others were retrospective studies. AHD was reported in 26 studies, of which 10 studies defined CD4 cell count and AIDS-defining diseases, 7 studies only defined CD4 count, and 9 studies only defined AIDS-defining diseases. LP was reported as an outcome of interest in 11 studies, of which 7 studies defined CD4 cell count and AIDS-defining diseases, and 4 studies only defined CD4 count. Among studies/arms presenting AHD, the time lag between initial HIV diagnosis and AIDS diagnosis or between HIV testing and first reported CD4 cell count was reported in 9 studies/arms as “at time of HIV diagnosis” (referred as 0 month), 3 studies/arms as one month, 8 studies/arms as three months (including 1 study as 60 days and 1 study as 90 days), 4 studies as six months, and 7 studies/arms as twelve months, respectively. Among studies presenting LP, the time lag between initial HIV diagnosis and AIDS diagnosis or between HIV testing and first reported CD4 cell count was reported in 7 studies/arms as “at time of HIV diagnosis” (including 1 study as 15 days), 3 studies/arms as three months (including 1 study as 90 days), 2 arms as twelve months, respectively.

The selected studies were published between 2002 and 2014. The number of participants per study/arm on advanced HIV diseases ranged from 113 to 28382, for a total of 1.55 million participants across 29 arms (63734 incident cases of AHD). The number of participants per study/arm on late presentation ranged from 352 to 5545, for a total of 48923 participants across 12 arms (29754 incident cases of LP).Nine studies were conducted in the United States, 8 in Italy, 3 in Spain, 2 in France, 2 in Mexico, and the other 10 studies were conducted in the following countries or region: Hong Kong, Thailand, India, Vietnam, Germany, Nigeria, Republic of Korea, Belgium. The percentage of females ranged from 9.00% to 70.88%. The *aORs* were reported in all studies. All risk measures were adjusted for confounding factors with number of adjusted variables ranging from 2 to 16.

### Risk of AHD or LP for males compared with females

**Figure 2:**
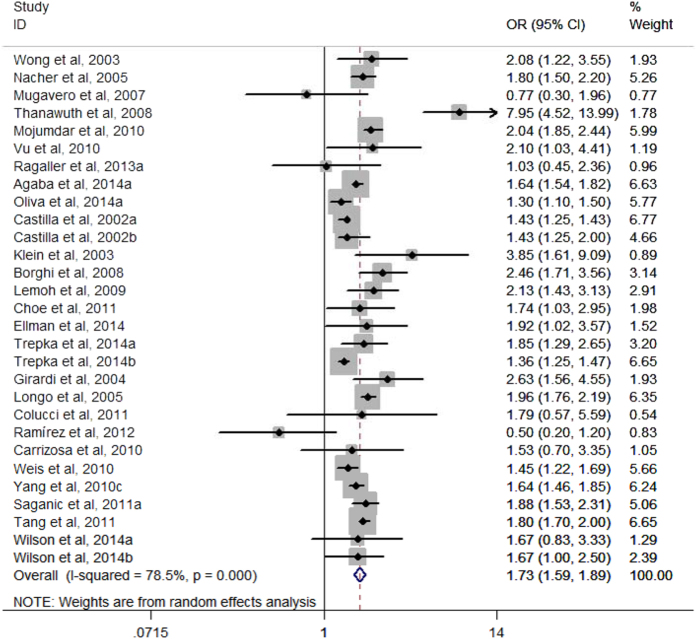
Forest plot of advanced HIV disease risk for males compared with females.

**Figure 3:**
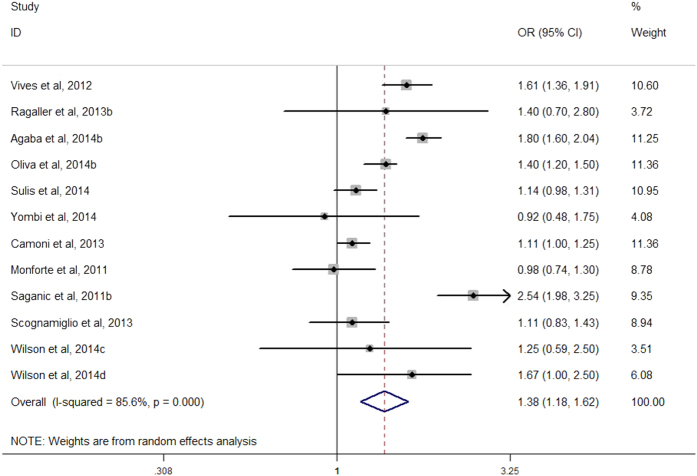
Forest plot of late presentation risk for males compared with females.

**Figure 4:**
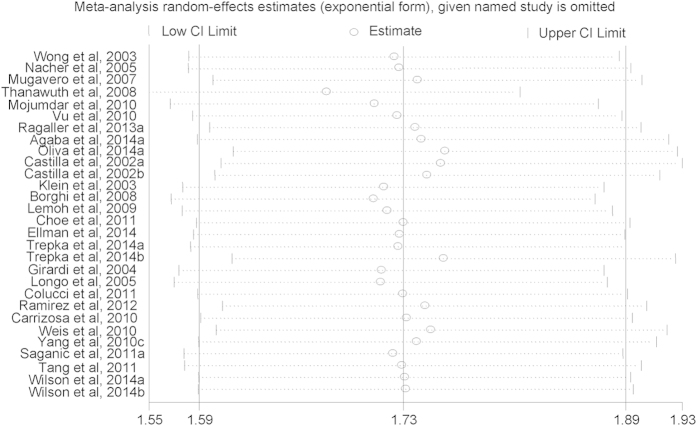
Sensitivity analysis of advanced HIV disease risk for males compared with females.

**Figure 5:**
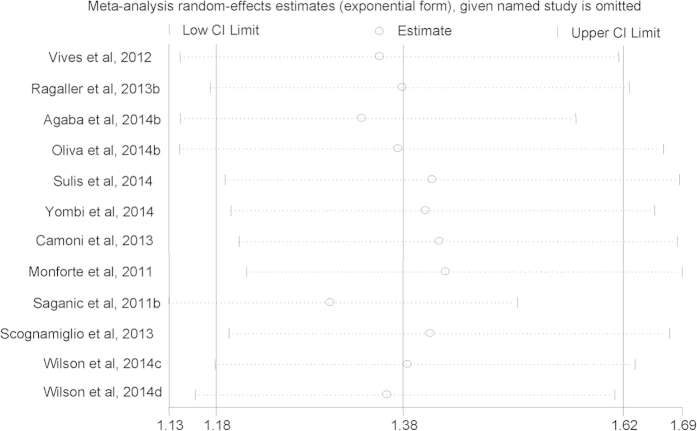
Sensitivity analysis of late presentation risk for males compared with females.

Among the 29 arms in 26 selected studies on AHD (Yang *et al.*, 2010a and Yang *et al.*, 2010b were excluded to calculate the pooled *aOR* due to shorter time lag), all but two found higher risk of presenting with AHD for males compared with females, although not all were statistically significant. Males had an increased risk of presenting with advanced HIV disease compared with females, with a pooled *aOR* of 1.73 (*95*% *CI*, 1.59–1.89) (Fig. 2). Among the 12 arms in 11 selected studies on LP, all but two found higher risk of presenting with late presentation for males compared with females, although not all were statistically significant. Males had an increased risk of presenting with LP compared with females, with a pooled *aOR* of 1.38 (*95*% *CI*, 1.18–1.62) (Fig. 3). A sensitivity analysis was conducted by omitting one study each time and re-calculating the pooled results for the remaining studies yielded consistent results. The overall risk of estimates did not vary materially ranging from 1.68 (*95*% *CI*, 1.55–1.82) to 1.76 (*95*% *CI*, 1.61–1.93) for studies on AHD (Fig. 4), and 1.30 (*95*% *CI*, 1.13–1.51) to 1.43 (*95*% *CI*, 1.21–1.69) for studies on LP (Fig. 5).

### Subgroup analysis

To explore the between-study heterogeneity, we performed subgroup analyses and meta-regression across a number of key study characteristics (Table 1). The finding of increased AHD or LP risk in males compared with females was consistently found in all of the subgroup analyses. The pooled risk of AHD in males compared with females of different time lags were *aOR* of 1.85 (*95*% *CI*, 1.50–2.30) with 0 month, *aOR* of 1.53 (*95*% *CI*, 1.33–1.76) with 1 month, *aOR* of 1.72 (*95*% *CI*, 1.47–2.02) with 3 month, *aOR* of 1.67 (*95*% *CI*, 0.97–2.67) with 6 month, and *aOR* of 1.70 (*95*% *CI*, 1.59–1.82) with 12 month, respectively. The pooled risk of LP in males compared with females of different time lags were *aOR* of 1.36 (*95*% *CI*, 1.14–1.62) with 0 month, *aOR* of 1.41 (*95*% *CI*, 0.77–2.58) with 3 month, and *aOR* of 1.54 (*95*% *CI*, 1.04–2.26) with 12 month, respectively. The selected study characteristics did not seem to significantly influence the results (*P* > 0.05), and only partly explained the source of heterogeneity.

### Publication Bias

**Figure 6:**
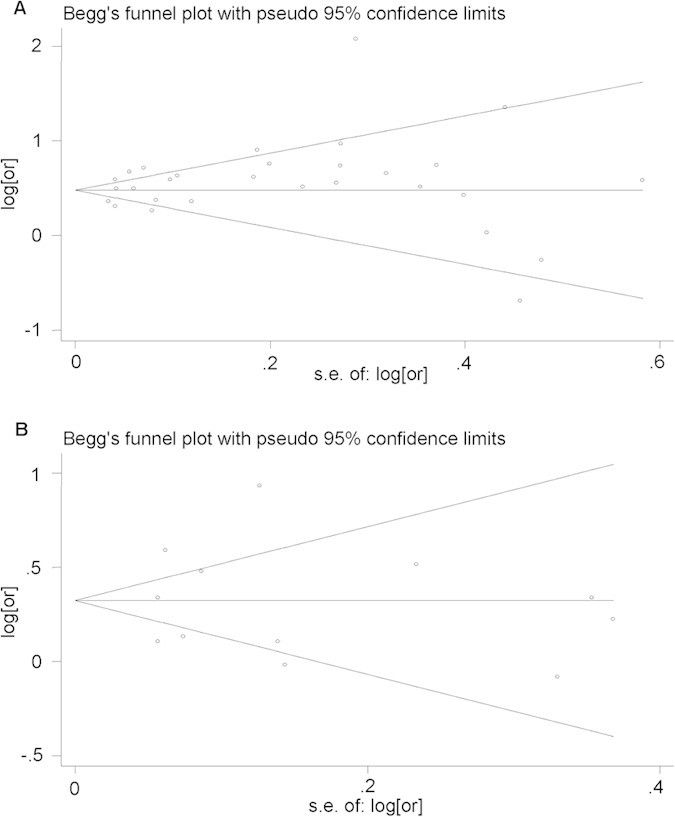
Funnel plots for publication bias of advanced HIV disease and late presentation. (**a**) Funnel plot for publication bias of advanced HIV disease; (**b**) Funnel plot for publication bias of late presentation.

No publication bias was observed in studies on AHD (Begg’ test: *z* = −0.49, *P* = 0.626; Egger’s test: *bias* = 0.87, *t* = 1.47, *P* = 0.153) or LP (Begg’ test: *z* = 0.14, *P* = 0.89; Egger’s test: *bias* = 0.01, *t* = 0.01, *P* = 0.996). Visual inspection of the Begg funnel plot revealed mostly symmetrically distribution confirming the absence of publication bias (Fig. 6).

## Discussion

There is an extensive body of literature reporting on the association between gender and the risk of late presentation and advanced HIV disease. The 32 studies that we identified report *aORs* which indicate higher risk of AHD and LP for males compared with females in all but 4 studies. Furthermore, the association persists and remains statistically significant across a number of subgroup analyses exploring selected study characteristics. The overall risk of estimates did not vary materially through sensitivity analysis, and no publication bias was shown in the current study. Given this consistency, we believed that these data provided concrete evidence that males are more likely to present with AHD or be LP compared with females.

Previous studies presented that HIV-infected women should experience a more favorable course of disease than men, and was corroborated by the fact that women tended to have higher CD4^+^ lymphocyte counts than men^29^. Studies also showed that women had lower plasma HIV-1 RNA values than men in the early years after acquisition of HIV-1 infection, but that these differences tended to disappear over time^30^,^31^. Less likely to be late presenter for women could be attributed to higher utilization of Voluntary Counseling and Testing services as part of routine health care services, and HIV testing was more widely accessible to women due to prenatal HIV screening, family planning, as well as gynecological follow-up^26^,^32^. A previous study presented that patients diagnosed of HIV for antenatal screening or having an HIV-positive sexual partner had higher CD4 cell count than patients with tuberculosis or HIV-related symptoms^33^. Another study showed that the risks of late entry to HIV care (defined as having CD4 T cell count 350 cells/μl or less and/or WHO clinical stage 3/4 documented within 3 months of enrollment) among men and women not pregnant were 74% and 52% higher than that among pregnant women, respectively^34^. However, the time of implementation of routine antenatal HIV screening varied across countries and regions, and so did the coverage rate of routine antenatal HIV screening because of different attitudes to HIV screening and other factors^35^,^36^. The highly specialized reference centers offering routine antenatal HIV screening could play an important role in providing HIV-infected pregnant women with optimal care and in reducing mother-to-child transmission rates to very low levels^37^. Further, a large proportion of newly HIV-infected women, being sexual partners of men with HIV-positive or at risk of acquiring HIV, had a higher perception of risk themselves^38^. Females got tested soon after their spouse test HIV positive, thereby getting tested at an earlier stage of disease before developing symptoms than their male counterparts. Moreover, more self perception of illness and need to access health care in females as compared to males, might result in higher chance of late presentation in males^39^,^40^. The association between gender and late diagnosis could also be influenced by more prevalent among males for a longer period than among females^41^, lower levels of HIV testing within certain male subgroups, some of whom may actually be MSM but had not been reported as such^42^,^43^,^44^.

It is estimated that as many as 21%–30% of HIV-infected individuals in the Europe and North America^45^,^46^,^47^ are currently unaware of their serostatus, and 10%–68% enter care and initiate treatment at a later disease stage than recommended^48^,^49^. Otherwise, over 20 different definitions have been used for a late presenter^50^. The inconsistent definition makes it difficult to conduct cross-country comparisons, assess the potential impact of different public health interventions to encourage earlier HIV diagnosis. A common definition of late presentation would be helpful to identify risk factors for late presentation in a common way, and hence identify whether risk factors vary in different settings. It would also simplify analyses that monitor changes in the rate of late presentation over time^12^. Thus the European Late Presenter Group (ELPG) proposed a consensus definition of late presentation based on CD4 count or clinical symptoms: “Persons presenting for care with a CD4 count below 350 cells/μL or presenting with an AIDS-defining event, regardless of the CD4 cell count” in 2011. In addition, the ELPG defined presentation with advanced HIV disease as “Persons presenting for care with a CD4 count below 200 cells/μL or presenting with an AIDS-defining event, regardless of CD4 cell count”^23^.

However, the proposed definitions did not specify the time lag between initial diagnosis of HIV infection and AIDS. Then Michael Kozak *et al.* suggested a 3-month time lag as an appropriate balance between missing data with a tighter window (for example, 30 days) and misclassification attributable to other health care processes with a longer window (for example, 12 months)^51^. Results from subgroup analysis in the current study also suggested that the time lag was a potential source of heterogeneity of the pooled results, though it was not significant through meta-regression. More studies to explore the appropriate time lag are still needed to facilitate better evaluation the issue of late presentation and advanced HIV disease. Many of studies were conducted in the developed countries/region, only a small number of studies in the developing countries. The socio-economic status and health policy varied from country/region to country which might in part explain the heterogeneity of the pooled results. When stratified by the number of adjusted variables, heterogeneity was reduced in the minimal subgroup (<4 variables), revealing that the number of adjusted variables could also be a potential source of heterogeneity. After stratifying by number of patients, the smaller number of patients subgroup (<1000 patients) showed lower heterogeneity (*I*^*2*^ = 68.70%, *P* < 0.001) for AHD and no heterogeneity (*I*^*2*^ = 0.00%, *P* = 0.526) for LP, possibly because more complex confounding factors were introduced into larger number of patients. Difference in data source and method of collecting data among cross-sectional study, retrospective study, and prospective study, might contribute to heterogeneity as well. More studies included higher proportion of males, and the different sex ratio of patients might cause heterogeneity of the pooled risk of advanced HIV disease or late presentation for males compared with females. Taken together, the result of subgroup analysis revealed that time lag, study location, number of patients, proportion of females, study design, number of adjusted variables might constitute source of heterogeneity, but only partly explained the heterogeneity.

To the best of our knowledge, this is the first meta-analysis to quantify the risk of AHD and LP for males compared with females. Advantages of the current study are applying European consensus definitions of AHD and LP and stratifying the results by varied time lags between initial diagnosis of HIV infection and AIDS. However, several limitations should be noted. To begin with, significant heterogeneity was observed in overall and subgroup analyses. Otherwise, the observation of heterogeneity should not reduce the confidence in the finding but just add some uncertainty about the magnitude of that^52^. Second, the majority of the studies included in the current study were retrospective studies, which depended on surveillance data, thus the integrity of data and possible recall bias might affect the results. What’s more, it may be a source of bias to combine data from different study designs^53^. Third, English and Chinese written literatures were searched and only English written literatures were included in the meta-analysis, which could have resulted in language bias, although previous studies suggest that this has little effect on the overall conclusions^54^,^55^. Last but not the least, the inclusion of only published articles which contained *aOR* may be a source of publication bias, despite the fact that no evidence of publication bias was exhibited by Begg’s or Egger’s tests.

## Conclusion

In conclusion, the current meta-analysis indicates that males are at higher risk of AHD or LP compared with females. Considering the consistent findings of a number of studies, and the reliability and robustness of our meta-analysis, we strongly recommend that more attention should be paid to males, more effort should be made to encourage individuals with high-risk behavior to participate counseling and testing, in order to make sure early testing, early diagnosis, and early treatment, ultimately to improve individual and population health.

## Method

### Search strategy

This meta-analysis was conducted in accordance with the Meta-Analysis of Observational Studies in Epidemiology guidelines. Pubmed, Embase, and Web of Science databases were searched for English-language publications dated until April 30th, 2015. Keywords used in the databases search included: Search Search (((((((gender) OR sex) OR males) OR females) OR men) OR women)) AND ((((((((((“HIV testing”) AND late)) OR ((“delayed diagnosis”) AND HIV)) OR “late HIV diagnosis”) OR ((“late presenters”) AND HIV)) OR “advanced HIV disease”) OR ((“late diagnosis”) AND HIV)) OR ((“late presentation”) AND HIV)) OR ((“late testers”) AND HIV)). Chinese Scientific Journals Fulltext Database (CQVIP), China National Knowledge Infrastructure (CNKI) and Wanfang Data were searched for Chinese-language publications dated until April 30th, 2015. Keywords used in the databases search included: (“Ai Zi Bing (AIDS)” OR “HIV”) and (“Jian Ce (Testing)” OR “Zhen Duan (Diagnosis)”). References lists of the retrieved articles and previous systematic reviews were also reviewed by researchers.

### Study Selection

Two authors examined the titles and abstracts of all studies containing keywords independently for eligibility. Studies were included based on the following criteria: (1) reported original data; (2) described “LP” as presence of an AIDS condition or CD4 cell count <350 cells/μL at presentation for care, or “AHD” to as the presence of either an AIDS condition or a CD4 cell count <200 cells/μL at presentation^12^,^23^, and (3) presented the estimates of the *aOR*, or *aRR* or adjusted *Hazard Ratio* (*aHR*) with the corresponding *95*% *CI* of gender difference in “LP” or “AHD”. Studies were excluded if they did not specified the exact duration between initial HIV diagnosis and AIDS diagnosis. If multiple publications reported results based on the same research, the more recent or complete article was included.

### Data Extraction

Data extraction was carried on by two authors independently using a pre-designed data extraction form. The following information was extracted from the included publications: last name of the first author, publication year, study period, study population, study location, outcome definition, time lag (duration between initial HIV diagnosis and AIDS diagnosis), data source, study design, number of patients, number of cases (patients with late presentation or advanced HIV disease), *aOR* or *aRR* or *aHR* with the corresponding *95*% *CI*, and adjusted variables. Number of patients and cases was the number of subjects included in the model of multivariate analysis.

### Statistical Methods

The adjusted estimates with the corresponding *95*% *CI* of gender difference in risk of AHD or LP were used to calculate the pooled estimates based on weighted pooled measures. If multiple arms using varies time lags in one study, arm with the longest time lag was included to calculate the overall pooled estimates. Forest plots were used to visually assess the individual and pooled estimates with the corresponding *95*% *CI*, and heterogeneity among studies was assessed using the Cochrane *Q* statistic significant when *P* < 0.10 and the *I*^*2*^ statistic^56^. The random-effect model was performed if *P* < 0.10 and *I*^*2*^ < 50%^57^. Sensitivity analysis was applied by excluding one study in turn to detect the influence of individual study on the pooled result. The subgroup analyses were also performed according to variables which might explain the potential source of heterogeneity. Meta-regression was conducted to assess the effects of selected factors on AHD or LP risk. Begg’ test and Egger’s test were used to assess the effect of publication bias^58^,^59^. Funnel plots were created used to evaluate potential publication bias using the standard error^60^. All statistical analyses were conducted using Stata Version 11.0 (StataCorp, College Station Texas). *P* < 0.05 was considered significant, except where otherwise specified.

## Additional Information

**How to cite this article**: Jiang, H. *et al.* Gender difference in advanced HIV disease and late presentation according to European consensus definitions. *Sci. Rep.* **5**, 14543; doi: 10.1038/srep14543 (2015).

## Supplementary Material

Supplementary Information

## Figures and Tables

**Table 1:** Subgroup analysis of advanced HIV disease and late presentation.

Subgroup analysis	Total No.		Pooled *aORs(95*%*CI)*	*I*^*2*^	*P* value								
	Studies/arms	Patients			Heterogeneity	Meta-regression							
Advanced HIV disease	29	155378	1.73(1.59–1.89)	78.50%	<0.001	—							
Time lag^a^													
0 month	9	34072	1.85(1.50–2.30)	84.90%	<0.001	0.754							
1 month	3	36544	1.53(1.33–1.76)	70.80%	0.033								
3 months	8	40841	1.72(1.47–2.02)	68.10%	0.003								
6 months	4	18394	1.67(0.97–2.76)	70.30%	0.018								
12 months	7	45455	1.70(1.59–1.82)	12.40%	0.335								
Study location													
developed countries/region	22	134907	1.67(1.53–1.82)	73.80%	<0.001	0.347							
developing countries	7	20471	1.98(1.47–2.69)	85.90%	<0.001								
Number of patients													
<1000	15	6711	1.92(1.44–2.55)	68.70%	<0.001	0.180							
≥1000	14	148667	1.65(1.52–1.79)	82.80%	<0.001								
Proportion of females^b^													
<50%	25	138309	1.76(1.59–1.94)	81.20%	<0.001	0.839							
≥50%	3	16791	1.67(1.54–1.80)	0.00%	0.683								
Study design													
cross-sectional	7	2257	1.63(0.82–3.23)	84.00%	<0.001	0.760							
retrospective	22	153121	1.70(1.57–1.84)	76.90%	<0.001								
Adjusted variables													
<4	5	25457	1.92(1.73–2.14)	0.00%	0.436	0.731							
=4	10	70400	1.69(1.47–1.95)	70.30%	<0.001								
>4	14	59521	1.73(1.52–1.96)	81.70%	<0.001								
Late presentation	12	48923	1.38(1.18–1.62)	85.60%	<0.001	—							
Time lag^c^													
0 month	7	41876	1.36(1.14–1.62)	85.60%	<0.001	0.706							
3 month	3	6254	1.41(0.77–2.58)	93.50%	<0.001								
12 months	2	793	1.54(1.04–2.26)	85.60%	<0.001								
Study location													
developed countries	11	34436	1.34(1.14–1.57)	81.50%	<0.001	0.317							
developing countries	1	14487	1.80(1.59–2.03)	—	—								
Number of patients													
<1000	4	1500	1.35(1.100–1.83)	0.00%	0.526	0.797							
≥1000	8	47423	1.40(1.16–1.68)	90.60%	<0.001								
Proportion of females^d^													
<50%	9	33736	1.34(1.13–1.59)	85.20%	<0.001	0.463							
≥50%	2	14839	1.78(1.58–2.01)	0.00%	0.329								
Study design													
cross-sectional	2	793	1.54(1.04–2.26)	0.00%	0.507	0.600							
retrospective	9	46395	1.40(1.17–1.68)	89.10%	<0.001								
prospective	1	1735	1.11(0.85–1.46)	—	—								
Adjusted variables													
<4	3	5767	1.40(0.99–1.31)	0.00%	0.684	0.121							
=4	6	24150	1.33(1.13–1.55)	72.10%	0.003								
>4	3	19006	1.66(1.08–1.62)	92.00%	<0.001								

^a^Time lag of 1 month included Yang *et al.*, 2010a and time lag of 3 months included Yang *et al.*, 2010b and one study as 60 days and one study as 90 days.

^b^Ragaller *et al.*, 2013a was excluded due to data not applicable.

^c^Time lag of 3 months included one study as 90 days.

^d^Ragaller *et al.*, 2013b was excluded due to data not applicable.
