# Supplementary material for: Gender difference in advanced HIV disease and late presentation according to European consensus definitions
Source: Sci Rep. 2015 Sep 28;5:14543. doi: 10.1038/srep14543 (PMC4585954; doi:10.1038/srep14543)
Supplement: Supplementary Information [file srep14543-s1.doc]

**Supplementary information**

**Gender difference in advanced HIV disease and late presentation according to European consensus definitions**

Hongbo Jiang, Jieyun Yin, Yunzhou Fan, Jianhua Liu, Zhixia Zhang, Li Liu, Shaofa Nie*

Department of Epidemiology and Biostatistics, School of Public Health, Tongji Medical College, Huazhong University of Science and Technology, 430030 Wuhan, Hubei, P.R. China,

^*^ **Corresponding author:**

Shaofa Nie, Department of Epidemiology and Biostatistics, School of Public Health, Tongji Medical College, Huazhong University of Science and Technology, No.13 Hangkong Road, Qiaokou District, Wuhan, Hubei, P.R. China.

Tel: +8602783693763; fax: +8602783693763; e-mail: [sf_nie@mails.tjmu.edu.cn](mailto:sf_nie@mails.tjmu.edu.cn).

Table S1 Study characteristics of studies included in the meta-analysis

| Study/Arm | Study Year | Study Population ^a^ | Study Location | Outcome Definition ^b^ | Time Lag | Data source | Study Design | No. Patients | No. Cases | *aOR(95%CI)* | Adjusted Variables |
| --- | --- | --- | --- | --- | --- | --- | --- | --- | --- | --- | --- |
| Wong et al,  2003 ^1^ | 1984-2000 | Female: 20.72% | Hong Kong | CD4 cell count of <200/μL at diagnosis | 0 month | Surveillance | Retrospective | 1530 | 540 | 2.08(1.22-3.55) | Ethnicity, ≥35 years of age at HIV diagnosis, Exposure category, |
| Nacher et al,  2005 ^2^ | 1992-2003 | Female: 51.95% | France | less than 200 CD4 lymphocytes/mm^3^ | 0 month | French Hospital Database for HIV | Retrospective | 1952 | 594 | 1.80(1.50-2.20) | Nationality, age, HAART availability |
| Mugavero et al, 2007 ^3^ | 2002-2004 | Mean age: 36.1±10 (range 17-61); Female: 27.4% | USA | An initial CD4 T lymphocyte count <200 cells/μL | 0 month | University-based HIV clinic | Cross-sectional | 113 | 55 | 0.77(0.30-1.96) | Age, race, uninsured, non-metropolitan |
| Thanawuth et al, 2008 ^4^ | 2004-2005 | Median age: 30 (IQR: 25-34); Female: 49% | Thailand | HIV-related symptoms at the time of first positive test | 0 month | seven public hospitals | Cross-sectional | 402 | 221 | 7.95(4.52-13.99) | Age at HIV diagnosis, employment status, primary risk factor, history of STD, Source of care for general illness, Perception of HIV-risk |
| Mojumdar et al, 2010 ^5^ | 2001-2007 | Female: 29.4% | India | CD4 T+ cell count below 200 cells/uL or with chronic clinical symptoms | 0 month | Retrospective data | Retrospective cross sectional | 3680 | 2936 | 2.04(1.85-2.44) | Age Group, Transmission route |
| Vu et al,  2010 ^6^ | 2004-2005 | Age range 18-54, Female: 19.1% | Vietnam | CD4 cell count of <200/mm^3^ at the time of the first positive test. | 0 month | Hospital for Tropical Diseases | Retrospective | 204 | 119 | 2.10(1.03-4.41) | Age, Injection drug use, Sexual contact, Opportunistic infection |
| Ragaller et al, 2013a ^7^ | 1986-2010 | Not Applicable | Germany | CD4 T cell counts less than 200/μl and/or clinical AIDS | 0 month | Three official HIV health care centres | Retrospective cohort study | 278 | 118 | 1.03(0.45-2.36) | Age groups, Years, risk groups |
| Oliva et al,  2014a ^8^ | 2007-2011 | Median age: 35 (IQR: 29-43); Female: 19% | Spain | CD4 count below 200 cells/μL in the first analysis performed after HIV diagnosis | 0 month | Surveillance | Retrospective | 11426 | 3356 | 1.30(1.10-1.50) | Age, Transmission mode, Region of origin, Year of diagnosis |

Table S1 Study characteristics of studies included in the meta-analysis (continued)

| Study/Arm | Study Year | Study Population | Study Location | Outcome Definition | Time Lag | Data source | Study Design | No. Patients | No. Cases | *aOR(95%CI)* | Adjusted Variables |
| --- | --- | --- | --- | --- | --- | --- | --- | --- | --- | --- | --- |
| Agaba et al,  2014a ^9^ | 2005-2010 | Median age: 33 (range 15-88); Female: 66.0% | Nigeria | CD4 count <200 cells/μL or AIDS-defining event, regardless of the CD4 count | 0 month | Jos University Teaching Hospital (JUTH) adult HIV clinic | Retrospective cohort study | 14487 | 9127 | 1.64(1.54-1.82) | Age, Marital status, Education, Occupation, HIV risk category, Source of referral, HBV coinfection, HCV coinfection |
| Castilla et al, 2002a ^10^ | 1994-1996 | Age>14: Female: 19.53% | Spain | First positive HIV test in the month of or immediately preceding AIDS diagnosis. | 1 month | Surveillance | Retrospective | 18755 | 4522 | 1.43(1.25-1.43) | Age, transmission category, prison record, country of origin, province of residence |
| Castilla et al, 2002b ^10^ | 1998-2000 | Age>14; Female: 20.92% | Spain | First positive HIV test in the month of or immediately preceding AIDS diagnosis. | 1 month | Surveillance | Retrospective | 7825 | 2758 | 1.43(1.25-2.00) | Age, transmission category, prison record, country of origin, province of residence |
| Yang et al,  2010a ^11^ | 2000-2007 | Age>12; Female: 27.5% | USA | AIDS diagnosis within one months of initial HIV diagnosis | 1 month | Surveillance | Retrospective | 9964 | 2740 | 1.73(1.52-1.96) | Race, Mode of transmission, Age group, Country of origin, Type of facility |
| Klein et al,  2003 ^12^ | 1998 | Age>17; Female: 14.95% | USA | CD4 cell count of <200/ L at diagnosis | 60 days | Medical records | Retrospective | 388 | 165 | 3.85(1.61-9.09) | Age group at diagnosis, Race/ethnicity, HIV risk group, Prior negative HIV test, Who requested HIV test, Time between first clinical indicator and testing positive for HIV |
| Borghi et al,  2008 ^13^ | 1992-2006 | Median age: 33.6 (IQR: 28.8-41.5); Female: 31.5% | Italy | CD4+ cell count <200 cells per microliter or AIDS within 3 months of their HIV-positive test. | 3 months | Surveillance | Retrospective | 844 | 332 | 2.46(1.71-3.56) | Age, Years of diagnosis, Exposure categories, Country of birth |

Table S1 Study characteristics of studies included in the meta-analysis (continued)

| Study/Arm | Study Year | Study Population | Study Location | Outcome Definition | Time Lag | Data source | Study Design | No. Patients | No. Cases | *aOR(95%CI)* | Adjusted Variables |
| --- | --- | --- | --- | --- | --- | --- | --- | --- | --- | --- | --- |
| Lemoh et al,  2009 ^14^ | 1994-2006 | Mean age: 37 (range 0-80); Female: 9.0% | USA | CD4 count below 200 cells/ mm^3^ at HIV diagnosis or diagnosis of AIDS earlier than 3 months after HIV diagnosis | 3 months | Surveillance | Retrospective | 2779 | 627 | 2.13(1.43-3.13) | Remoteness, Region of birth, Exposure to HIV, Age at HIV diagnosis, Year of HIV diagnosis |
| Yang et al,  2010b ^11^ | 2000-2007 | Age>12; Female: 27.5% | USA | AIDS diagnosis within 3 months of initial HIV diagnosis | 3 months | Surveillance | Retrospective | 9964 | 3178 | 1.47(1.33-1.62) | Race, Mode of transmission, Age group, Country of origin, Type of facility |
| Choe et al,  2011 ^15^ | 1987-2008 | Median age: 36 (IQR: 29-45); Female: 9% | Republic of Korea | CD4 cell count of less than 200 cells/mm^3^ within 3 months of HIV diagnosis. | 3 months | Medical center | Retrospective | 994 | 405 | 1.74(1.03-2.95) | Age, HIV Transmission risk, Syphilis serostatus, Year of Presentation |
| Ellman et al,  2014 ^16^ | 2006-2011 | Female: 29.6% | USA | Concurrent with AIDS-Defining Illness or CD4 Count <200 Cells/μL | 90 days | Medical center | Retrospective | 287 | 121 | 1.92(1.02-3.57) | Age， Race/ethnicity， Risk factor，Prior medical visits |
| Trepka et al,  2014a ^17^ | 2007-2011 | Rural; Age>12; Female: 33.11% | USA | AIDS diagnosis within 3 months of HIV diagnosis | 3 months | Surveillance | Retrospective cohort study | 746 | 267 | 1.85(1.29-2.65) | Year of HIV diagnosis, Age group at diagnosis, Race/ethnicity, Country of birth, Mode of HIV transmission, Percent of population in ZCTA, Number of doctors in county |

Table S1 Study characteristics of studies included in the meta-analysis (continued)

| Study/Arm | Study Year | Study Population | Study Location | Outcome Definition | Time Lag | Data source | Study Design | No. Patients | No. Cases | *aOR(95%CI)* | Adjusted Variables |
| --- | --- | --- | --- | --- | --- | --- | --- | --- | --- | --- | --- |
| Trepka et al,  2014b ^17^ | 2007-2011 | Urban; Age>12; Female: 27.40% | USA | AIDS diagnosis within 3 months of HIV diagnosis | 3 months | Surveillance | Retrospective cohort study | 24839 | 6793 | 1.36(1.25-1.47) | Year of HIV diagnosis, Age group at diagnosis, Race/ethnicity, Country of birth, Mode of HIV transmission, Percent of population in ZCTA, Number of doctors in county |
| Girardi et al,  2004 ^18^ | 1997-2000 | Female: 23.84% | Italy | A first HIV-positive test in the 6 months preceding study enrollment and they had a CD4 count <200/mm^3^ or clinically defined AIDS at enrollment | 6 months | Italian Cohort Naive Antiretrovirals | Multicenter, longitudinal observational study | 713 | 280 | 2.63(1.56-4.55) | Year of enrollment, Geographic area, Age, Education, Employment status, Having children, Negative HIV test before the first positive, Counseling at the first positive test, Use of injecting drugs, Use of noninjecting drugs, Number of sexual partners in the previous month, Same gender sex |
| Longo et al,  2005 ^19^ | 1996-2002 | Age≥18; Female: 23.68% | Italy | first positive HIV test result within six months of the AIDS diagnosis | 6 months | Surveillance | Retrospective | 17007 | 6253 | 1.96(1.76-2.19) | Age, Area of origin, Exposure category, Year of AIDS diagnosis |
| Ramírez et al,  2012 ^20^ | 2001-2008 | Mean age: 35 (range 28-74); Female: 11% | Mexico | AIDS defining event (ADE) or developed it in the following six months, had an initial CD4+ cell count lower than 200/mm^3^ or both. | 6 months | HIV Clinic | Cross-sectional | 429 | 264 | 0.50(0.20-1.20) | Age, Married, Transmission route, Unemployed, Socioeconomic Status, Education, Period |

Table S1 Study characteristics of studies included in the meta-analysis (continued)

| Study/Arm | Study Year | Study Population | Study Location | Outcome Definition | Time Lag | Data source | Study Design | No. Patients | No. Cases | *aOR(95%CI)* | Adjusted Variables |
| --- | --- | --- | --- | --- | --- | --- | --- | --- | --- | --- | --- |
| Colucci et al,  2011 ^21^ | 2003-2005 | Age≥18,mean age: 42.3±8.7; Female: 17.55% | Italy | With a time period of 6 months between first HIV positive test and AIDS diagnosis. | 6 months | Centers of infectious diseases | Cross-sectional | 245 | 127 | 1.79(0.57-5.59) | Age, Area of origin, Education, Being employed, Mode of acquisition of infection, Place where the first HIV test was performed, Reasons for HIV testing, Who suggested HIV test, At least one symptom or illness, Hospitalization 12 months before the first HIV test, Occasional partners 12 months before HIV diagnosis, Paid sexual intercourses 12 months before HIV diagnosis |
| Weis et al,  2010 ^22^ | 2001-2005 | Age≥18, mean age: 37.5±11.1; Female: 32.6% | USA | AIDS diagnosis within 1 year of their first positive HIV test | 12 months | Surveillance | Retrospective | 4137 | 1791 | 1.45(1.22-1.69) | Residence, Race/ethnicity, Exposure, Age |
| Yang et al,  2010c ^11^ | 2000-2007 | Age>12; Female: 27.5% | USA | AIDS diagnosis within 12 months of initial HIV diagnosis | 12 months | Surveillance | Retrospective | 9964 | 3597 | 1.64(1.46-1.85) | Race, Mode of transmission, Age group, Country of origin, Type of facility |
| Tang et al,  2011 ^23^ | 2002-2006 | Age≥13; Female: 13.17% | USA | diagnosed with HIV within 12 months before their AIDS diagnosis | 12 months | Surveillance | Retrospective | 28382 | 17364 | 1.80(1.70-2.00) | Age group (in years) at HIV diagnosis, Risk factor, Race/ethnicity and country of birth, Initial AIDS diagnosis |

Table S1 Study characteristics of studies included in the meta-analysis (continued)

| Study/Arm | Study Year | Study Population | Study Location | Outcome Definition | Time Lag | Data source | Study Design | No. Patients | No. Cases | *aOR(95%CI)* | Adjusted Variables |
| --- | --- | --- | --- | --- | --- | --- | --- | --- | --- | --- | --- |
| Carrizosa et al, 2010 ^24^ | Not Applicable | Mean age: 34.12±9.26 (range: 18-69); Female: 30.2% | Mexico | (1) an AIDS-defining illness within 1 year of first positive HIV test; (2) a date of AIDS diagnosis within 1 year of first positive HIV test; or (3) an initial CD4 cell count below 200 cells per microliter within 1 year of first positive HIV test. | 12 months | HIV/AIDS public clinics | Cross-sectional | 275 | Not Applicable | 1.53(0.7-3.35) | Clinic, Age at HIV diagnosis, Marital status, Years of education, Earned income, HIV exposure category, Alcohol/drug use before sex, Condom use, Type of regular healthcare provider, Type of HIV tester, Peers with high-risk sexual practices, Peers with HIV infection, Exposure to preventive information, Site of first positive HIV test, Stigma re: HIV-infected people, Stigma re: testing, Delayed testing because... |
| Saganic et al, 2011a ^25^ | 2000-2008 | Age≥18; Female: 14.81% | USA | AIDS within 12 months of initial HIV diagnosis. | 12 months | Surveillance | Retrospective | 1904 | 704 | 1.88(1.53-2.31) | Race/ethnicity, risk category, age at HIV diagnosis, residence in King County, and foreign-born status |
| Wilson et al,  2014a ^26^ | 2003-2011 | Migrants born in SSA; Female: 70.88% | France | <200 CD4 cells/mm^3^ or an AIDS defining event within the calendar year of diagnosis | 12 months | National representative survey in Metropolitan France | Cross-sectional | 352 | 143 | 1.67(0.83-3.33) | Age at diagnosis, Educational attainment, Time between migration and diagnosis, Religiosity |
| Wilson et al,  2014b ^26^ | 1999-2011 | Female: 44.22% | France | <200 CD4 cells/mm^3^ or an AIDS defining event within the calendar year of diagnosis | 12 months | National representative survey in French overseas departments | Cross-sectional | 441 | 164 | 1.67(1.00-2.5) | Age at diagnosis, Educational attainment, Cohabiting couple |

Table S1 Study characteristics of studies included in the meta-analysis (continued)

| Study/Arm | Study Year | Study Population | Study Location | Outcome Definition | Time Lag | Data source | Study Design | No. Patients | No. Cases | *aOR(95%CI)* | Adjusted Variables |
| --- | --- | --- | --- | --- | --- | --- | --- | --- | --- | --- | --- |
| Vives et al,  2012^27^ | 2001-2008 | Age>13, median age: 35 (IQR: 29-43); Female: 22.5% | Spain | CD4 cell count <350 cells/mL or with an AIDS-defining condition at presentation. | 0 month | Surveillance | Retrospective | 4651 | 2598 | 1.61(1.36-1.91) | Year of diagnosis, Age categories, Country of birth, Exposure categories |
| Ragaller et al, 2013b ^7^ | 1986-2010 | Not Applicable | Germany | CD4 T cell counts less than 350/μl and/or clinical AIDS | 0 month | Three official HIV health care centres | Retrospective cohort study | 348 | 188 | 1.40(0.70-2.80) | Age groups, Years, risk groups |
| Agaba et al,  2014b ^9^ | 2005-2010 | Median age: 33 (range 15-88); Female: 66.0% | Nigeria | CD4 count <350 cells/μL or AIDS-defining event, regardless of the CD4 count | 0 month | Jos University Teaching Hospital (JUTH) adult HIV clinic | Retrospective cohort study | 14487 | 12401 | 1.80(1.60-2.04) | Age, Marital status, Education, Occupation, HIV risk category, Source of referral, HBV coinfection, HCV coinfection |
| Oliva et al,  2014b ^8^ | 2007-2011 | Median age: 35 (IQR: 29-43); Female: 19% | Spain | CD4 count below 350 cells/μL in the first analysis performed after HIV diagnosis | 0 month | Surveillance | Retrospective | 11426 | 5494 | 1.40(1.20-1.50) | Age, Transmission mode, Region of origin, Year of diagnosis |
| Sulis et al,  2014 ^28^ | 2000-2010 | Age≥18; Female: 27.89% | Italy | a CD4+T cell count<350/μl and/or presenting with an AIDS-defining clinical condition at baseline | 0 month | Hospitals or university units | Retrospective | 5060 | 2413.00 | 1.14(0.98-1.31) | Mode of transmission, Age, Country of origin |
| Yombi et al,  2014 ^29^ | 2007-2011 | Female: 28.69% | Belgium | CD4 count <350/mm^3^ at the time of diagnosis | 0 month | Surveillance | Retrospective | 359 | 154 | 0.92(0.48-1.75) | Age, Origin, HIV transmission |

Table S1 Study characteristics of studies included in the meta-analysis (continued)

| Study/Arm | Study Year | Study Population | Study Location | Outcome Definition | Time Lag | Data source | Study Design | No. Patients | No. Cases | *aOR(95%CI)* | Adjusted Variables |
| --- | --- | --- | --- | --- | --- | --- | --- | --- | --- | --- | --- |
| Camoni et al,  2013 ^30^ | 2010-2011 | Female: 25.16% | Italy | CD4 ≤350 cells/μL or diagnosed with AIDS (regardless of the CD4 cell count) | 15 days | Surveillance | Retrospective | 5545 | 3059 | 1.11(1.00-1.25) | Age at diagnosis, Nationality, Area of notification, Transmission mode |
| Monforte et al, 2011 ^31^ | 2007-2009 | Median age: 36(IQR: 31-41); Female: 25.40% | Italy | A diagnosis of AIDS or CD4+ T-cell count at baseline≤350/mm^3^ | 3 months | Surveillance | Retrospective cohort study | 2276 | 838 | 0.98(0.74-1.30) | Mode of transmission, Age, nationality, hepatitis coinfection, calendar year of HIV test per more recent year, viral load per log higher, level of education, employment status |
| Saganic et al, 2011b ^25^ | 2000-2009 | Age≥18; Female: 15.07% | USA | The initial CD4+ T-cell count is <350 cells/mL | 90 days | Surveillance | Retrospective | 2243 | 1256 | 2.54(1.98-3.25) | Race/ethnicity, risk category, age at HIV diagnosis, residence in King County, and foreign-born status |
| Scognamiglio et al, 2013 ^32^ | 2004-2009 | Median age: 36 (range 18-86); Female: 21.60% | Italy | CD4 count <350 cells/mm^3^ or an AIDS-defining event within 3 months of HIV diagnosis | 3 months | Surveillance | Prospective multi-centre observational | 1735 | 890 | 1.11(0.83-1.43) | Age, area of birth, HIV exposure category and type of indicator disease |
| Wilson et al,  2014c ^26^ | 2003-2011 | Migrants born in SSA; Female: 70.88% | France | <350 CD4 cells/mm^3^ or an AIDS defining event within the calendar year of diagnosis | 12 months | National representative survey in Metropolitan France | Cross-sectional | 352 | 226 | 1.25(0.59-2.5) | Age at diagnosis, Educational attainment, Time between migration and diagnosis, Religiosity |
| Wilson et al,  2014d ^26^ | 1999-2011 | Female: 44.22% | France | <350 CD4 cells/mm^3^ or an AIDS defining event within the calendar year of diagnosis | 12 months | National representative survey in French overseas departments | Cross-sectional | 441 | 237 | 1.67(1.00-2.5) | Age at diagnosis, Department, Educational attainment, Cohabiting couple |

Note: No. of patients (people living with HIV/AIDS) and cases (individuals with advanced HIV disease or late presentation) were number of study population included in the logistic regression model. *aOR* is short for adjusted *odds ratio* and *95%CI* for *95% confidence interval*.

^a^ IQR is short for inter-quartile range; SSA is short for Sub-Sahara Africa.

^b^ The HIV-related symptoms included AIDS-defining illnesses or opportunistic infections; The chronic clinical symptoms included fever, diarrhea, weight loss etc.

**References**

1. Wong, K. H., Lee, S. S., Low, K. H. & Wan, W. Y. Temporal trend and factors associated with late HIV diagnosis in Hong Kong, a low HIV prevalence locality. *AIDS Patient Care STDS* **17**, 461-469 (2003).

2. Nacher, M. *et al.* Risk factors for late HIV diagnosis in French Guiana. *AIDS* **19**, 727-729 (2005).

3. Mugavero, M. J., Castellano, C., Edelman, D. & Hicks, C. Late diagnosis of HIV infection: the role of age and sex. *Am J Med* **120**, 370-373 (2007).

4. Thanawuth, N. & Chongsuvivatwong, V. Late HIV diagnosis and delay in CD4 count measurement among HIV-infected patients in Southern Thailand. *AIDS Care* **20**, 43-50 (2008).

5. Mojumdar, K., Vajpayee, M., Chauhan, N. K. & Mendiratta, S. Late presenters to HIV care and treatment, identification of associated risk factors in HIV-1 infected Indian population. *BMC Public Health* **10**, 416 (2010).

6. Nhac-Vu, H. T., Giard, M., Phong, N. D. & Vanhems, P. Risk factors for delayed HIV diagnosis at the Hospital of Tropical Diseases in Ho Chi Minh City, Vietnam. *Int J STD AIDS* **21**, 802-805 (2010).

7. Spornraft-Ragaller, P., Boashie, U., Stephan, V. & Schmitt, J. Analysis of risk factors for late presentation in a cohort of HIV-infected patients in Dresden: positive serology for syphilis in MSM is a determinant for earlier HIV diagnosis. *Infection* **41**, 1145-1155 (2013).

8. Oliva, J. *et al.* Predictors of advanced disease and late presentation in new HIV diagnoses reported to the surveillance system in Spain. *Gac Sanit* **28**, 116-122 (2014).

9. Agaba, P. A. *et al.* Patients who present late to HIV care and associated risk factors in Nigeria. *HIV Med* **15**, 396-405 (2014).

10. Castilla, J. *et al.* Late diagnosis of HIV infection in the era of highly active antiretroviral therapy: consequences for AIDS incidence. *AIDS* **16**, 1945-1951 (2002).

11. Yang, B. *et al.* Late HIV diagnosis in Houston/Harris County, Texas, 2000-2007. *AIDS Care* **22**, 766-774 (2010).

12. Klein, D., Hurley, L. B., Merrill, D. & Quesenberry, C. P., Jr. Review of medical encounters in the 5 years before a diagnosis of HIV-1 infection: implications for early detection. *J Acquir Immune Defic Syndr* **32**, 143-152 (2003).

13. Borghi, V. *et al.* Late presenters in an HIV surveillance system in Italy during the period 1992-2006. *J Acquir Immune Defic Syndr* **49**, 282-286 (2008).

14. Lemoh, C. *et al.* Delayed diagnosis of HIV infection in Victoria 1994 to 2006. *Sex Health* **6**, 117-122 (2009).

15. Choe, P. G. *et al.* Late presentation of HIV disease and its associated factors among newly diagnosed patients before and after abolition of a government policy of mass mandatory screening. *J Infect* **63**, 60-65 (2011).

16. Ellman, T. M., Sexton, M. E., Warshafsky, D., Sobieszczyk, M. E. & Morrison, E. A. A forgotten population: older adults with newly diagnosed HIV. *AIDS Patient Care STDS* **28**, 530-536 (2014).

17. Trepka, M. J. *et al.* Late HIV diagnosis: Differences by rural/urban residence, Florida, 2007-2011. *AIDS Patient Care STDS* **28**, 188-197 (2014).

18. Girardi, E. *et al.* Delayed presentation and late testing for HIV: demographic and behavioral risk factors in a multicenter study in Italy. *J Acquir Immune Defic Syndr* **36**, 951-959 (2004).

19. Longo, B., Pezzotti, P., Boros, S., Urciuoli, R. & Rezza, G. Increasing proportion of late testers among AIDS cases in Italy, 1996-2002. *AIDS Care* **17**, 834-841 (2005).

20. Crabtree-Ramirez, B., Caro-Vega, Y., Belaunzaran-Zamudio, F. & Sierra-Madero, J. High prevalence of late diagnosis of HIV in Mexico during the HAART era. *Salud Publica Mex* **54**, 506-514 (2012).

21. Colucci, A. *et al.* Characteristics and behaviors in a sample of patients unaware of their infection until AIDS diagnosis in Italy: a cross-sectional study. *AIDS Care* **23**, 1067-1075 (2011).

22. Weis, K. E., Liese, A. D., Hussey, J., Gibson, J. J. & Duffus, W. A. Associations of rural residence with timing of HIV diagnosis and stage of disease at diagnosis, South Carolina 2001-2005. *J Rural Health* **26**, 105-112 (2010).

23. Tang, J. J., Levy, V. & Hernandez, M. T. Who are California's late HIV testers? An analysis of state AIDS surveillance data, 2000-2006. *Public Health Rep* **126**, 338-343 (2011).

24. Carrizosa, C. M. *et al.* Determinants and prevalence of late HIV testing in Tijuana, Mexico. *AIDS Patient Care STDS* **24**, 333-340 (2010).

25. Saganic, L. *et al.* Comparing Measures of Late HIV Diagnosis in Washington State. *AIDS Res Treat* **2012**, 182672 (2012).

26. Wilson, K. *et al.* Frequency and correlates of late presentation for HIV infection in France: older adults are a risk group - results from the ANRS-VESPA2 Study, France. *AIDS Care* **26 Suppl 1**, S83-93 (2014).

27. Vives, N. *et al.* Factors associated with late presentation of HIV infection in Catalonia, Spain. *Int J STD AIDS* **23**, 475-480 (2012).

28. Sulis, G. *et al.* Clinical and epidemiological features of HIV/AIDS infection among migrants at first access to healthcare services as compared to Italian patients in Italy: a retrospective multicentre study, 2000-2010. *Infection* **42**, 859-867 (2014).

29. Yombi, J. C. *et al.* Late presentation for human immunodeficiency virus HIV diagnosis results of a Belgian single centre. *Acta Clin Belg* **69**, 33-39 (2014).

30. Camoni, L., Raimondo, M., Regine, V., Salfa, M. C. & Suligoi, B. Late presenters among persons with a new HIV diagnosis in Italy, 2010-2011. *BMC Public Health* **13**, 281 (2013).

31. d'Arminio Monforte, A. *et al.* Late presenters in new HIV diagnoses from an Italian cohort of HIV-infected patients: prevalence and clinical outcome. *Antivir Ther* **16**, 1103-1112 (2011).

32. Scognamiglio, P. *et al.* The potential impact of routine testing of individuals with HIV indicator diseases in order to prevent late HIV diagnosis. *BMC Infect Dis* **13**, 473 (2013).
